# Dust Exposure, Fractional Exhaled Nitric Oxide and Respiratory Symptoms among Volcanic Rock Miners in Kilimanjaro, Tanzania

**DOI:** 10.29024/aogh.2320

**Published:** 2018-08-31

**Authors:** Simon Mamuya, Gloria Sakwari, Vera Ngowi, Bente Moen, Magne Bråtveit

**Affiliations:** 1 Muhimbili University of Health and Allied Sciences, Environmental and Occupational Health Department, Dar es Salaam, TZ; 2 Center for International Health, University of Bergen, NO; 3 Department of Global Public Health and Primary Care, University of Bergen, NO

## Abstract

Volcanic rock for use as building material is mined extensively in the North Eastern Region of Tanzania. Dust emitted from the rock may contain harmful elements such as crystalline silica, arsenic (As), cobalt (Co), boron (B) and mercury (Hg) which might contribute to severity and onset of health symptoms.

**Objective:**

This study assessed respiratory symptoms and fractional exhaled nitric oxide as a marker for respiratory inflammation in relation to dust exposure among workers in different job sections in volcanic block mining.

**Materials and Methods:**

A cross- sectional study assessed a total of 135 workers in which 70 were exposed and 65 none exposed. The mining activities are mainly manual, and include cutting of blocks underground, transporting blocks to the shaping area, shaping blocks, loading blocks and aggregates (Murom) to vehicles and clearing or expanding the site. Respiratory health questionnaires were administered through face–to-face interviews. A total of 28 samples of “total” dust were collected around the breathing zone of the workers using SKC Sidekick pump (model 224–50) with a flow rate of 2.0 l/min. FE_NO_ assessed respiratory system inflammation using a portable electrochemistry-based sensor (NIOX MINO).

**Findings:**

The overall arithmetic mean concentration of personal total dust exposure among the workers was 4.37 mg/m^3^ (range 0.15-20.84). The prevalence of acute cough and red eyes were significantly higher among exposed than among non exposed (35% vs 10% and 45% vs 14%, respectively). The ANOVA Boniferroni test showed a significant difference in mean FENO between stone cutters and none exposed (P = 0.005).

**Conclusions:**

This study suggests the strong association between working as a stone cutting and shaping with respiratory inflammation. There is a need for respiratory mask type P2 use to protect workers from the exposure. There is also need for the follow up study involving cohorts of all workers happened to be in the mine.

## Introduction

Occupational exposure to dust is a major cause of respiratory symptoms and diseases among miners [1,2,3,4,5,6]. Volcanic rock for use as building material is mined extensively in the North-eastern region of Tanzania. Workers at the mining sites cut rocks manually to an approximate size of 5” × 6” × 20” before it is transported to the surface for further shaping by hand tools. Activities associated with dust exposure include clearing the underground way, underground block cutting, shaping, transport to the surface and loading of blocks and aggregates to vehicles. The final rock building material comprises aggregates, stones and blocks. Dust emitted from the materials may contain harmful elements such as crystalline silica, arsenic (As), cobalt (Co), boron (B) and mercury (Hg), which might contribute to severity and onset of health symptoms [7,8]. Occupational effects expected in rock mining include respiratory symptoms, decreased lung functions, silicosis and chronic obstructive pulmonary disease (COPD). The severity of the problem, among other factors, depends on the exposure duration, the composition and the concentration of the dust.

A study by Mamuya and coworkers in 2006 in a Tanzanian coal mine, showed that workers were affected differently according to their job category [9]. The development workers had more respiratory symptoms than workers in other sections, as they drilled through hard rock containing high concentration of crystalline silica [10]. South African studies have also showed high prevalence of respiratory symptoms among coal mine workers [11], and progression of respiratory symptoms were noted even after miners had stopped working in underground gold and iron ore mines [1,12]. Most studies in mines show that respiratory symptoms and decreased lung function correlate well with cumulative exposure [22], which takes into account both exposure intensity and duration. Cigarette smoking and age appeared as the greatest confounders for the association between dust exposure and respiratory health [11].

However, studies of respiratory effects related to dust exposure among volcanic block miner have, to our knowledge, not been done. This study aimed to assess respiratory symptoms and fractional exhaled nitric oxide as a marker for respiratory inflammation among workers in different job sections in volcanic block mining in Kilimanjaro area, Tanzania.

## Materials and Methods

A cross-sectional study was conducted among volcanic building material miners in the areas of Rongoma, Pumwani, Kawawa, Kiuo B, Uchira and Urenga hills in Kilimanjaro region. The mining activities are mainly manual, and include underground cutting of blocks, transporting blocks to the shaping area, shaping blocks, loading blocks and aggregates (Murom) to the vehicle and clearing or developing the site. The volcanic building material is composed of sedimentary rock originated thousands of years ago from of volcanoes on the slopes of Mount Kilimanjaro.

## Study subjects

A total of 70 workers involved in volcanic stone cutting were included in the study with 44 controls from working as casual workers in a drink factory where there is no dust exposure as a result of work activities and 21 from the community around. Out of those, 70 volcanic stone workers were include in the assessment of acute symptoms while only 21 controls were included in the assessment. The workers from the miners were those involved in block cutting and shaping and transportation, and the controls were 44 workers from a soft drink factory involved in loading, unloading of crates in and 21 from the community who wereschool teachers, post policemen, village leaders and women supplying food to the area. Volcanic block miners who had been in the job for more than a year were eligible for the study. Due to intermittent availability and mobility, the workers loading vehicles were not studied, and since there was no development of new mining sites in the study period, no workers involved in clearing the sites were included.

## Personal dust exposure

A total of 28 samples of “total” dust were collected around the breathing zone of the workers using SKC Sidekick pump (model 224-50) with a flow rate of 2.0 l/min. Dust were collected on cellulose acetate filters (pore size 0.8 μm) placed in 37 mm closed-faced Millipore cassettes, which were assembled and labeled at Muhimbili Laboratory in Dar es Salaam. Sampling started immediately before the start of the morning shift and lasted for two to four hours. Five samples were taken every day in the 14 days. The 28 dust samples were analyzed gravimetrically in the Muhimbili laboratory using a Mettler Toledo AT 261 delta range with accuracy of 0.05 mg.

## Respiratory health questionnaire

Respiratory health questionnaires were administered to workers through face-to-face interviews. The questionnaire consisted of three parts, including personal and work characteristics, chronic and acute respiratory health symptoms and smoking habits [4]. The questions on personal and work characteristics included sex, age, height, education level, employment history, years worked in mine, years in dusty work elsewhere and previous diseases.

A modified version of the British Medical Research Council questionnaire was used for chronic respiratory symptoms [13]. The questionnaire was prepared in English and was translated into Kiswahili, the national language of Tanzania before it was used.

The questionnaire included questions on cough, breathlessness and wheezing. The responses required were either ‘yes’ or ‘no’ for having symptoms or not having symptoms, respectively. Questions on cough were whether a person experienced cough with or without sputum production in the morning, day and night, cough as much as 4–6 times a day for four or more days in a week and cough for most of days for as much as three consecutive months or more in a year. Questions on breathlessness were based on whether a person experienced breathlessness when hurrying on level ground or walking up slight hill, walking with other people of their own age on level ground and stopping for breath when walking at their own pace on level ground. Questions on wheezing were whether a person had attacks of wheezing in the chest at any time, and the duration of that wheezing. The participants were asked whether they had bronchial asthma and/or other chronic illnesses such as tuberculosis and bronchitis.

Questions on acute symptoms were elicited as whether a worker had the following symptoms, responding ‘yes’ or ‘no’ for dry cough, shortness of breath, wheezing, stuffy nose, runny nose, irritated nose and sneezing during or after the present working day.

Smoking status included questions on whether the worker was smoking at the time of the study or whether he had smoked more than one cigarette a day and stopped less than a year prior to the time of study (currently smoking), whether he had smoked previously and stopped more than a year ago (ex-smoker), the year they stopped and number of cigarettes smoked per day. For current smokers, the amount of cigarette packets smoked in a year was calculated by multiplying numbers of cigarettes smoked per day times duration of smoking in days divided by 20. Never smokers were defined as individuals who had never smoked [14]. Questions on other pulmonary diseases which might have confounded the results were elicited. A worker was asked whether he had injury or operation affecting the chest, whether he had any of the heart problems, bronchitis, pneumonia, pleurisy, pulmonary tuberculosis, bronchial asthma or any other chest problems in the past three years. Those found with any of the mentioned pulmonary problems were excluded from the analysis.

## Fractional exhaled nitric oxide measurement (FE_NO_)

All participants who were non-smokers were eligible to participate. Out of these there were 38 exposed workers and 39 controls involving 21 and 18 bachelor’s students on the occupational health module. We measured FE_NO_ using a portable electrochemistry-based sensor (NIOX MINO; Aerocrine AB, Solna, Sweden) according to ATS/ERS recommendations on online measurements of FE_NO_ [15], and the results were expressed as parts per billion. Only one measurement of FE_NO_ was taken per person, as studies have shown that one measurement is adequate using the NIOX MINO. The participants were asked to exhale, then inhale through the device and exhale steadily for 10 seconds at a flow rate of 50 ml/s and at a pressure of 10 cm H_2_O. The flow rate and pressure were automatically controlled and any wrong performance was automatically rejected by the device. The measurements were done in a sitting position. Room levels of nitric oxide were measured before the testing began, and it was below 5ppb. All participants were asked for the time since their last meal before the measurement was taken. Those who reported to have taken food less than an hour prior to the measurements were not subjected to test.

## Statistical analysis

The statistical package for social sciences (SPSS) version 11.5 was used for the data analysis. The probability value of 0.05 and less was chosen as the criterion for the statistical significance. The exposure data were skewed so it was log transformed for analysis. Analysis of variance (ANOVA) was used on the FENO data to compare means between different groups. Chi-square test was used to compare proportions in categorical variables (χ^2^). Logistic regression analyses were used to determine odds ratios (OR) for FENO categories (FENO < 25 ppb = 0 and FENO >= 25 = 1) while controlling for age and duration of employment. Multiple regression analysis was used to determine association between dust exposure and FENO, while adjusting for age and sex.

## Ethical clearance

Ethical clearance was obtained from the Muhimbili University of Health and Allied Sciences Research and Publication Committee. The research permits were obtained from Regional Commissioner, Kilimanjaro Region and Moshi District council and from the wards executive officers of the mine sites. The Northern zone mine administration was informed of the project and allowed the study to be conducted. Each person was informed on the aim of the study and requested to participate voluntarily. The informed consent was obtained orally.

## Results

The results show that the majority of the study population were male in both exposed workers and controls 62 (88%) and 53 (82%), respectively. The stone cutters were noted to have mean education years of about seven years for both exposed and controls. The prevalence of current smoking was 30.9% among exposed group and 0 among controls (p = 0.079) (Table 1).

**Table 1:** Socio demographic characteristics of study population.

Characteristics	Volcanic stone workers (n = 70)	Controls (n = 65)	

Age Mean(SD) (years)	33.6(12.3)	34.2(13.6)	
Education level, Mean(SD)	6.9(1.7)	9.4(2.9)	
Tenure Mean(SD) years)	7.8(6.7)	6.0(5.9)	
**Sex n (%)**			
Male	62(88.6)	53(81.5)	
Female	8(42.9)	12(18.5)	
**Smoking n (%)**			
Current smoker	21(30)	9(41)	*
Non smoker	49(70)	56(86)	

The overall arithmetic mean concentration of personal total dust exposure among the workers was 4.37 mg/m^3^ (range 0.15–20.84). Dust exposure was highest at the Pumwani mine site (9.00 mg/m^3^), and lowest at Uchira (0.57 mg/m^3^) (Table 2).

**Table 2:** Distribution of mean dust concentrations for workers in Rongoma, Pumwani, Kawawa and Uchira volcanic mines.

Place	N	Mean(SD)	Median	Minimum	Maximum

Rongoma	10	2.76(3.86)	1.14	0.39	12.23
Pumwani	10	9.00(5.83)	8.75	2.10	20.84
Kawawa Kiuo	5	0.63(0.44)	.63	0.15	1.20
Uchira	3	0.57(0.48)	.44	0.17	1.11
Total	28	4.37	1.44	0.15	20.84

The prevalence of all chronic respiratory symptoms was higher among exposed workers than among controls. The differences in cough day and night with sputum, wheezing, chest tightness and in dyspnoea when walking on uphill or running had significant differences between the groups (Table 3).

**Table 3:** Prevalence of Chronic respiratory symptoms by exposure status among volcanic miners.

Reported Symptoms	Exposed (n = 70)	Controls (n = 65)	p-value^#^

Morning cough	13(18.6)	4(6.2)	0.651
Cough day & night	21(30.0)	4(6.2)	0.058
Morning cough with sputum	10(14.3)	4(6.2)	0.038
Cough day & night with sputum	14(20)	2(3.1)	0.003
Chest tightness	17(24.6)	1(1.5)	0.001
Wheezing	20(28.6)	5(7.7)	0.002
Dyspnoea 1	21(30.0%)	3(4.6)	0.003
Dyspnoea 2	11(15.7%)	1(1.5)	0.014
Dyspnoea 3	7(10.0%)	0(0)	–

^#^ Fischer’s exact.

When the respiratory symptoms were adjusted for age, smoking habit and years at work, there was a significantly higher odds ratio among exposed workers for cough day and night (AOR (3.9: 95%CI (1.1–14.1))) (Table 7).

The exposed workers also had higher prevalence than controls for the all acute symptoms (Table 4). The prevalence of acute cough and red eyes were significantly higher among exposed than among controls (35% vs. 10% and 45% vs. 14%, respectively) (Table 4).

**Table 4:** Acute respiratory symptoms by exposure status among volcanic miners.

Reported Symptoms	Exposed (n = 69)	Controls (n = 21)	Total	p-value*	

Acute cough	23(34.8%)	2(10.0%)	25(29.1%)	0.032	*
Acute dyspnoea	13(18.8%)	1(4.8%)	14(15.6%)	0.131	*
Acute wheezing	12(17.4%)	2(9.5%)	14(15.6%)	0.612	
Sneezing	33(47.8%)	6(28.6%)	40(44.4%)	0.119	
Red eyes	31(44.9%)	3(14.3%)	35(38.9%)	0.011	
Itching eyes	28(41.2%)	4(19.0%)	32(36.0%)	0.174	

*Fischer’s exact test.

Acute cough and red eyes were the symptoms among volcanic building material found to be significantly different when compared to the control group with (p = 0.032 and 0.011, respectively). Other respiratory symptoms among miners were not significantly different from the control.

The exposed group had higher mean FENO compared to the control group (28.2 vs. 18.7 ppb, respectively). Analysis of work categories showed higher mean FENO among stone cutters (28.8 ppb) compared to transport workers (15.8 ppb) and controls (16.7 ppb) (Table 5). The ANOVA Boniferroni test showed a significant difference in mean FENO between stone cutters and controls (p = 0.005), while the difference between stone cutters and transport workers was borderline significant (p = 0.057). Multiple regression analysis model including FENO as a dependent variable showed that after adjustment for sex, the exposure status (1/0) contributed significantly in the model (p = 0.011), indicating that FENO is 9.1 ppb higher for exposed than controls (Table 6).

**Table 5:** Distribution of Exhaled nitrogen oxide among non-smokers study population by exposure status and gender.

Sex	Exposure status	Mean (SD) ppb	Work category	Mean (SD) ppb

Male	Exposed (n = 36)	27.89(19.14)	Stone cutter	28.8(19.59)
	Control (n = 25)	16.88(9.19)	Transportation	15.80(5.33)
			Control	16.70(8.53)
Female	Exposed (n = 2)	16.50(4.95)		
	Control (n = 14)	14.93(5.12)		
Total	Exposed (N = 38)	27.29(18.81)		
	Control (N = 39)	16.18(7.95)		

**Table 6:** Multiple regression analysis of the FeNO values by exposure status.

Model		Unstandardized Coefficients		Standardized Coefficients	Sig.	95% Confidence Interval for B		
	
		B	Std. Error	Beta		Lower Bound	Upper Bound

R^2^ = 13.5%	(Constant)	25.534	7.108		.001	11.410	39.658
	Age	–.082	.125	–.066	.510	–.330	.165
	Sex	–4.425	3.935	–.115	.264	–12.245	3.394
	Exposed and controls (1/0)	9.101	3.034	.309	.004	3.072	15.129

**Table 7:** Odds ratio for development of respiratory symptoms among volcanic stone cutters.

Symptoms	Odds Ratio (95%CI)	Adjusted Odds ratio	p-value

Morning cough	3.5(1.7–11.3)	1.8(0.5–7.0)	0.370
Day and Night	6.5(2.1–20.3)	3.9(1.3–14.1)	0.034
Wheezing	4.8(1.7–13.7)	3.1(0.9–10.0)	0.064

## Discussion

The result of this study revealed high total dust exposure levels among workers in the mine responsible for cutting and shaping of the blocks. This is similar to studies done in a Tanzanite gemstone mine in Mererani [21] and another study in a Tanzanian coal mine [9]. The high dust exposure levels depict the use of manual work in cutting of the rocks together with the use of blunt tools for shaping the blocks and the proximity of the workers’ breathing zone and the work tool. In addition no dust suppression measures such as water during cutting and shaping were used. A study among construction workers in Mozambique showed high dust emission from demolition work without water sprinkling, which caused silica dust to spread all over the surrounding environment [16]. The differences between exposures among sites were characterized by differences in the rock types.

The significant finding in our study is the higher fractional exhaled nitric oxide (FENO) among exposed workers in the mine sites when compared to control workers. This implies that being a volcanic block cutter increases one’s chance of increased FENO by about 9.1 ppb compared to controls. A study done by Moen et al. among coffee dust workers in the same geographical region showed similar trends among coffee workers, who had higher FENO levels than the control, thus indicating a relationship between exposure and lung inflammation [17]. Similar findings were also obtained in a study in cement workers in Norway, where the difference between shifts denoted a higher FENO increase among cement workers than control [18]. Another study showed the chronicity of the respiratory disease among asbestosis workers who had elevated FENO as compared to internal and external controls [19]. FENO levels below 25 ppb has been indicated to show normal respiratory inflammation while above 25 ppb is associated with increased bronchial inflammation, which can be associated by extrinsic factors such as dust or allergens [15].

The current study shows similar findings on respiratory symptoms to a study in coal mining in Tanzania, though the workers in the volcanic mine had more smokers (30.9%) compared to the coal mine workers (16.1%) [10]. However, the FENO measurements in the present study was assessed to among non-smoking study population. Like the study in the coal mine, the current study showed a strong association between acute respiratory symptoms with exposure to the volcanic dust. The symptoms being acute implies that they show up when a worker is on duty or soon after the work shift.

Chronic respiratory symptoms was another effect investigated in the current study, like in many other dust studies. Contrary to many studies, our study did not show any significant difference between exposed workers and the control for all the chronic symptoms tested. This anomaly might be explained by the small numbers among the control. The other explanation might be that those with chronic diseases were no longer fit for the job. The young age of the study population and the short exposure duration in the job might also explain the findings. It might be that the exposure is high but due to intermittent working on the volcanic block, they have not yet had adequate exposure for the dust to cause chronic respiratory problems. It was noted that working in the block making as a cutter or transporter is seasonally based, due to the fact that the population in the area are peasants with small farms and they work in their farm from January to March. They work in the mine in the season just after the farm harvest to get funds for sending children to school and end-of-year celebrations.

Inflammation of respiratory track system was elicited clearly by the respiratory symptom and confirmed by the FENO levels. The multiple regression analysis justify the FENO levels to be strongly associated with the volcanic dust exposures, the model explained 13.5% of variances. The analysis showed that working in the volcanic dust increased FENO by 9.1 ppb compared to the control workers.

This study suffers from a number of limitations, among them is the selection bias in which the study period could not allow the enumeration of all workers involved in the block cutting and shaping. The study just sampled only those who were present during the visit. It is obvious that some who were missing on that day might have some characteristics that might explain the study otherwise. The healthy workers’ effect might be another bias of this study, as the already sick workers have left the job. The small sample size is another that might have biased the study. Information bias might be another, since the data interviewers were research assistants being a number of environmental health students thoroughly trained and qualified to collect the data. The FENO measurement was taken by SHM, and the instrument does not record the unacceptable FENO by any standard. Workers were informed earlier on the day that they should not eat anything two hours before the measurement. The need for investigation on the type of the dominant elements might be of interest, as studies suggest this type of rocks contains very dangerous heavy metals [5,7,8,20].

Notwithstanding its limitations this study does suggest the strong association between working in stone cutting and shaping and respiratory inflammation. That implies control measures should be instigated among miners before the situation becomes worse. There is a need for immediate use of respiratory mask type P2 to protect them from the exposure and consequently prevent them from acquiring chronic respiratory conditions. There is a need for further studies involving cohorts of all workers who happen to be in the mine and current workers, with appropriate design to capture changes over a period.
